# Circulating miR-210 and miR-22 combined with ALT predict the virological response to interferon-alpha therapy of CHB patients

**DOI:** 10.1038/s41598-017-15594-0

**Published:** 2017-11-15

**Authors:** Jin Li, Xiaonan Zhang, Liang Chen, Zhanqing Zhang, Jiming Zhang, Weixia Wang, Min Wu, Bisheng Shi, Xinxin Zhang, Maya Kozlowski, Yunwen Hu, Zhenghong Yuan

**Affiliations:** 1 Research Unit, Shanghai Public Health Clinical Center, Fudan University, Shanghai, China; 2 Key Laboratory of Medical Molecular Virology at the School of Basic Medical Sciences, Shanghai Medical College, Fudan University, Shanghai, China; 3 Department of Hepatology, Shanghai Public Health Clinical Center, Fudan University, Shanghai, China; 4 Department of Infectious Diseases, Huashan Hospital, Fudan University, Shanghai, China; 5 Institute of Infectious and Respiratory Diseases, School of Medicine, Shanghai Jiaotong University, Ruijin Hospital, Shanghai, China

## Abstract

Interferon-alpha (IFN-α) therapy of chronic hepatitis B (CHB) patients is constrained by limited response and side effects. We described a panel of circulating microRNAs (miRNAs) which could potentially predict outcome of IFN-α therapy. Here, we report development of a simplified scoring model for personalized treatment of CHB patients. 112 CHB patients receiving IFN-α treatment were randomly divided into a training (n = 75) or a validation group (n = 37). The expression of 15 candidate miRNAs was detected in training group with 5 miRNAs exhibiting significantly different levels (p < 0.0001) between early virological response (EVR) and non-early virological response (N-EVR). These 5 miRNAs were further tested in validation phase. Refinement analyses of results from training phase established a model composed of miR-210, miR-22 and alanine aminotransferase (ALT), with area under ROC curve (AUC) of 0.874 and 0.816 in training and validation groups, respectively. In addition, this model showed prognostic value for sustained virological response (SVR) (AUC = 0.821). Collectively, this simplified scoring model composed of miR-210, miR-22 and ALT can reproducibly predict the EVR and SVR of IFN-α therapy in CHB patients. The model should help to forecast the outcome of IFN-α treatment prior to therapy decision involving nucleoside analogs or IFNs.

## Introduction

Hepatitis B is a major global health problem. Infection by the Hepatitis B virus (HBV) can lead to multiple stages of liver disease including asymptomatic HBV carrier state, chronic hepatitis B (CHB), liver cirrhosis, liver failure and hepatocellular carcinoma^1,2^. Hepatitis B prevalence is the highest in the Western Pacific Region and the African Region, where 6.2% and 6.1%, respectively of the adult populations are infected (WHO, Guidelines on hepatitis B and C testing, February 2017). According to the recent WHO estimates 248 million people are chronically infected with hepatitis B resulting in 887,000 deaths in 2015, mostly due to complications, including cirrhosis and hepatocellular carcinoma (WHO, Guidelines on hepatitis B and C testing, February 2017).

CHB infections are currently treated with two types of antiviral medications: interferon alpha (IFN-α)/PEGylated interferon-alpha (Peg-IFN-α) and nucleoside analogs^3^. Treatment of CHB patients with IFN-α/Peg-IFN-α increases the rate of hepatitis B surface antigen (HBsAg) clearance and improves the seroconversion rate of hepatitis B e antigen (HBeAg)^3–5^. However, favorable response to IFN-α treatment has only been achieved in a fraction of CHB patients^6^. Furthermore, the IFN-α therapy is frequently accompanied by significant side effects such as thrombocytopenia and leucopenia^7^. To avoid development of IFN-α mediated adverse effects and complications, it is paramount to pre-identify the CHB patients who would benefit from this therapy^8^.

It is well recognized that the host and viral factors, including baseline serum alanine aminotransferase (ALT), baseline HBV DNA, HBV genotype, patients’ gender and a dynamics of surface antigen titer may influence the sustained response to IFN-α treatment of CHB patients^9–12^. In view of such complexity, a discovery of novel biomarkers facilitating the prediction of IFN-α treatment outcomes is of paramount importance. Recent efforts have focused on identification of potential factors that could be used to forecast the outcome of IFN-α therapy. For example, microarray analysis of intrahepatic mRNA profiles identified a list of candidate genes linked to sustained response^8^. Likewise, a baseline level of serum interleukin-23 (IL-23) also showed a prognostic value^13^. Nevertheless, there is still a great need for development of novel biomarkers with higher prediction accuracy and convenient operation.

MicroRNAs (miRNAs) are a class of 19–24 nt RNA molecules that do not encode for any protein but are able to regulate gene expression in the body^14^. Since their discovery in serum and plasma^15^, considerable effort has been directed to the study of circulating miRNAs as biomarkers of diseases^16–19^. As biomarkers, circulating miRNA provide a number of advantages such as stability in extreme conditions, relative sequence conservation, time-space specificity of expression and simple measurement^20,21^. The presence of circulating miRNAs in serum and other body fluids which are known to contain ribonucleases, suggests that miRNAs are shielded from degradation by either packaging into lipid vesicles, forming complexes with RNA-binding proteins, or both^21^. Such protection would afford miRNAs stability in circulation.

Previously, we proposed a predictive model consisting of 11 miRNAs identified following microarray screening of 959 miRNAs in the plasma of 94 CHB patients who received IFN-α treatment^7^. The present study was undertaken with the aim to extend these earlier findings by further validating the prediction efficacy of identified plasma miRNAs and to construct a simplified scoring model, which could be used to improve personalized treatment of CHB patients.

## Results

### Clinical characteristics

**Figure 1:**
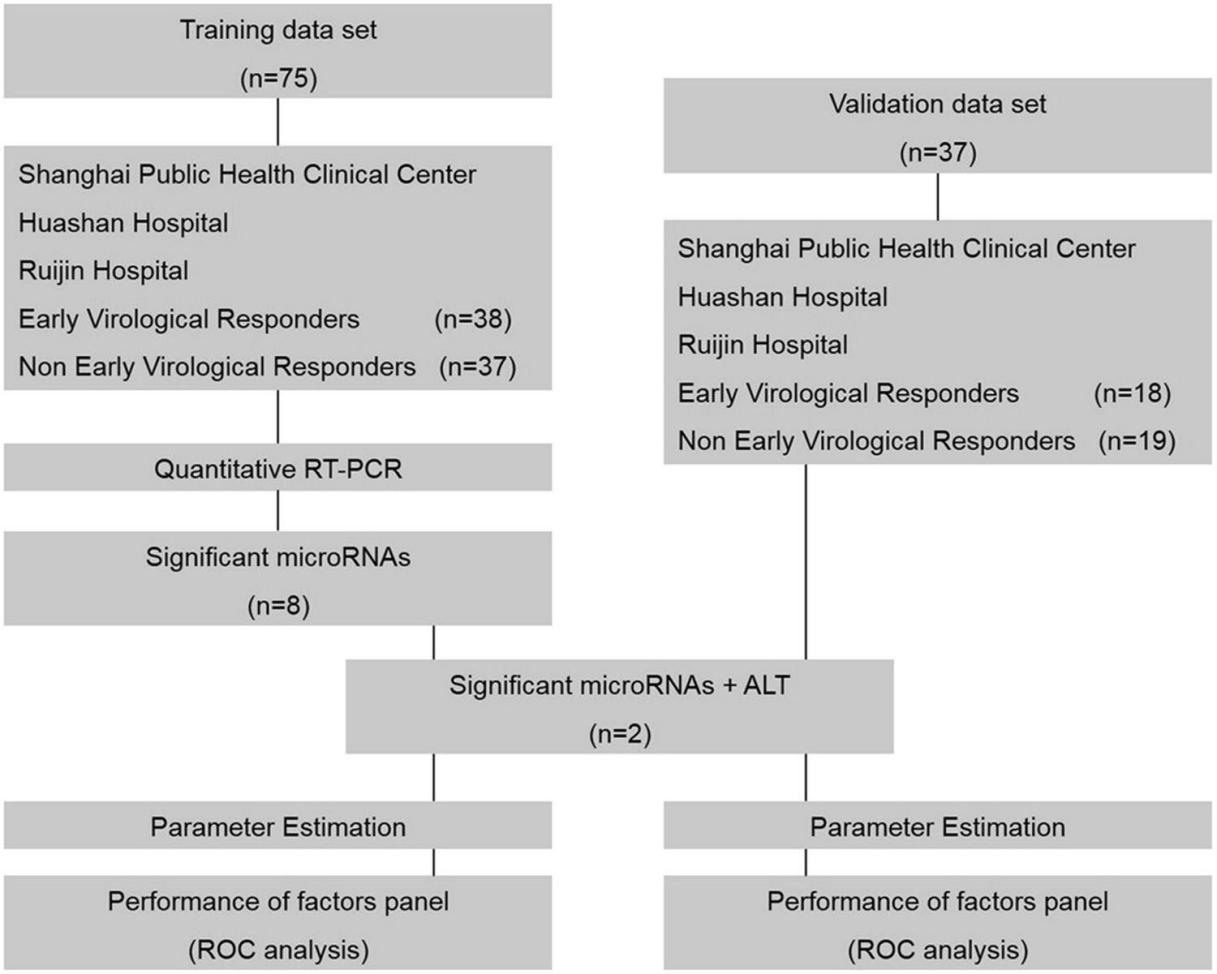
Study design. The 112 plasma samples were collected from CHB patients who received IFN-α therapy. These samples were allocated to training and validation phases in a chronological order. The samples were then examined for the levels of selected miRNAs and clinical markers of disease. Significant miRNAs and ALT were identified as predictive factors in both the training and validation phases. The best scores were subjected to the ROC curve analysis for the prediction of IFN-α therapy outcome in CHB patients. RT-PCR, reverse transcriptase polymerase chain reaction; ALT, alanine transaminase; ROC curve, receiver operating characteristics curve.

The baseline characteristics of patients in training and validation groups are shown in Table 1. The baseline clinical variables, including age, gender, genotype, baseline ALT, HBsAg and HBeAg levels, showed no statistical difference (p > 0.05; Table 1), while, the expression levels of HBV DNA displayed a slight difference between the two groups (P < 0.05; Table 1). On the whole, the basic characteristics of enrolled patients were comparable between these two groups (Fig. 1).

**Table 1:** Basic characteristics of enrolled patients at baseline.

Variables	Entire cohort	Training Phase	Validation Phase	P value
**No. of patients**	112	75	37	
**Age (year)**				0.9760
median	28	28	28.5	
range	16–63	16–55	20–63	
**Gender**				0.2119※
Male (%)	72 (64.3)	45 (60)	27 (73)	
Female (%)	40 (35.7)	30 (40)	10 (27)	
**Genotype**				0.3678※
B type (%)	31 (27.7)	17 (22.7)	14 (37.8)	
C type (%)	65 (58)	43 (57.3)	22 (59.5)	
D type (%)	2 (1.8)	2 (2.7)	0 (0)	
Missing (%)	14 (12.5)	13 (17.3)	1 (2.7)	
**ALT (U/L)**				0.7693
median	109	110	103.5	
range	44–645	45–645	44–636	
**HBV DNA (log**_**10**_ **copies/ml)**				0.0177
median	7.15	6.91	7.55	
range	4.12–8.24	4.12–8	5.32–8.24	
**HBsAg (log**_**10**_ **COI)**				0.2907
median	3.93	3.92	3.95	
range	2.33–4.06	2.33–4.06	3.1–4.06	
**HBeAg (log**_**10**_ **COI)**				0.5311
median	2.17	2.16	2.21	
range	−0.97–3.28	−0.84–3.28	−0.97–3.01	

Abbreviations: IU = International Units; COI = Cut Off Index; ALT = serum alanine aminotransferase; DNA = deoxyribonucleic acid; HBsAg = hepatitis B surface antigen; HBeAg = hepatitis B e antigen; The levels of HBV DNA, HBsAg and HBeAg were log10 transformed; P value: Comparison between training phase and validation phase (Mann Whitney test); Only genotype frequency of B and C were analyzed; ※Fisher’s exact test. Please note, that the patients were also screened for liver damage which showed variability in all groups.

All of the patients were categorized into EVR or N-EVR groups - based on whether a decrease of over 2 log10 of viral load at the 12th week was achieved (Table 2). In the training group, 37 patients (49.3%) showed EVR, and a similar trend was observed in the validation group where 19 patients (51.4%) exhibited EVR (Table 2, Fig. 1). It has been reported that some basic clinical indicators, such as the levels of HBsAg or HBeAg, can be used to predict the effect of IFN-α treatment^9,12,22^. We therefore aimed to identify the potential predictive factors among all the clinical variables, including age, gender, genotype, and levels of HBsAg, HBeAg and HBV DNA at baseline. We compared variability of these factors between N-EVR and EVR groups in both training and validation phases. Interestingly, ALT levels were significantly higher in the EVR groups in both the training (p = 0.0065; Table 2) and validation phases (p < 0.0001; Table 2). Other variables did not show statistical difference in either groups (Table 2, Supplementary Figure 1).

**Table 2:** The comparison of basic characteristics between N-EVR and EVR groups in the two phases.

Variables	Training Phase			Validation Phase		
	N-EVR	EVR	P value	N-EVR	EVR	P value
**Number, %**	38 (50.7%)	37 (49.3%)		18 (48.6%)	19 (51.4%)	
**Age (year)**			0.6121			0.3486
median	28	29		29	27	
range	16–55	20–55		23–63	20–43	
**Gender**			0.6410※			0.7140※
Male (%)	24 (63.2)	21 (56.8)		14 (77.8)	13 (68.4)	
Female (%)	14 (36.8)	16 (43.2)		4 (22.2)	6 (31.6)	
**Genotype**			0.3986※			0.3217※
B type (%)	6 (15.8)	11 (29.7)		5 (27.8)	9 (47.4)	
C type (%)	21 (55.3)	22 (59.5)		12 (66.7)	10 (52.6)	
D type (%)	2 (5.2)	0 (0)		0 (0)	0 (0)	
Missing (%)	9 (23.7)	4 (10.8)		1 (5.5)	0 (0)	
**ALT (U/L)**			0.0065			<0.0001
median	95	157.5		82	132	
range	45–270	50–645		44–202	74–636	
**HBV DNA (log**_**10**_ **copies/ml)**			0.1670			1.0000
median	6.72	7.18		7.50	7.56	
range	4.12–8	5.05–8		5.64–8	5.32–8.24	
**HBsAg (log**_**10**_ **COI)**			0.4334			0.1176
median	3.92	3.93		3.98	3.95	
range	2.33–4.06	3.15–4.01		3.51–4.06	3.10–4.00	
**HBeAg (log**_**10**_ **COI)**			0.8211			0.2545
median	2.20	2.13		2.28	1.94	
range	−0.84–3.21	−0.83–3.28		−0.83–3.01	−0.97–2.83	

Abbreviations: EVR = Early Virological Response, N-EVR = Non Early Virological Response; COI = Cut Off Index; ALT = serum alanine amino transferase; DNA = deoxyribonucleic acid; HBsAg = hepatitis B surface antigen; HBeAg = hepatitis B e antigen; The levels of HBV DNA, HBsAg and HBeAg were log10 transformed; P value: Comparison between training phase and validation phase (Mann Whitney test); Only genotype frequency of B and C were analyzed; ※Fisher’s exact test.

### Identification of significant predictive miRNAs

**Figure 2:**
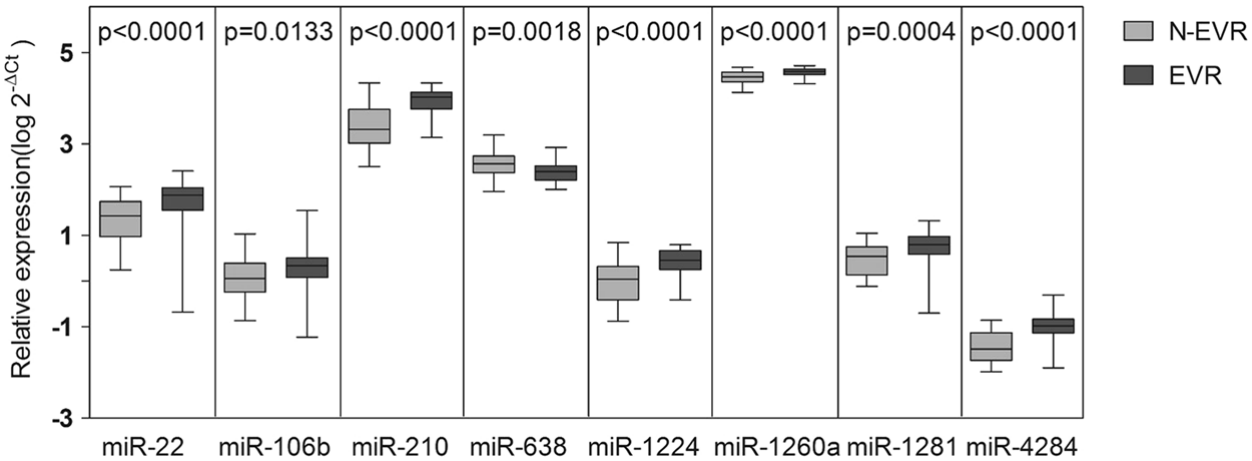
Expression levels of eight miRNAs selected in the training phase. The expression levels of miRNAs differentially expressed in the training phase were compared between 38 early virological responders (EVR; dark) and 37 non-early virological responders (N-EVR; grey) by RT-qPCR. The lines in boxes represent median values, and the deviation bars represent min to max values. P-values represent two-sided Mann Whitney test.

To identify additional biomarkers which would provide high prediction accuracy of IFN-α therapy, we analyzed 15 candidate miRNAs selected from the microarray screening of plasma samples from CHB patients as well as from the relevant published reports^7,23–26^ (Supplementary Table 1). The expression levels of these 15 miRNAs were compared between EVR (n = 37) and N-EVR (n = 38) groups in training phase (Supplementary Table 2). Eight miRNAs, including miR-22, miR-106b, miR-210, miR-638, miR-1224, miR-1260a, miR-1281, and miR-4284 exhibited higher expression levels in the EVR than in N-EVR group (P < 0.05; Fig. 2). Among them, the expression levels of miR-22, miR-210, miR-1224, miR-1260a, miR-4284 between EVR and N-EVR groups displayed more significant difference (P < 0.0001; Supplementary Table 2). Hence, they were qualified as significant miRNAs and included in the next step of analysis. Additionally, we further filtered these five miRNAs by their abundance, since miRNAs with lower quantity would be more prone to errors. Based on this criterion, miR-1224 and miR-4284 were excluded from further analysis since the median values of their relative expression levels were lower than 1. Following these analyses miR-22, miR-210 and miR-1260a were considered as significant predictive miRNAs and included in the further analysis.

### Establishing the predictive factors and validating the predictive model

**Figure 3:**
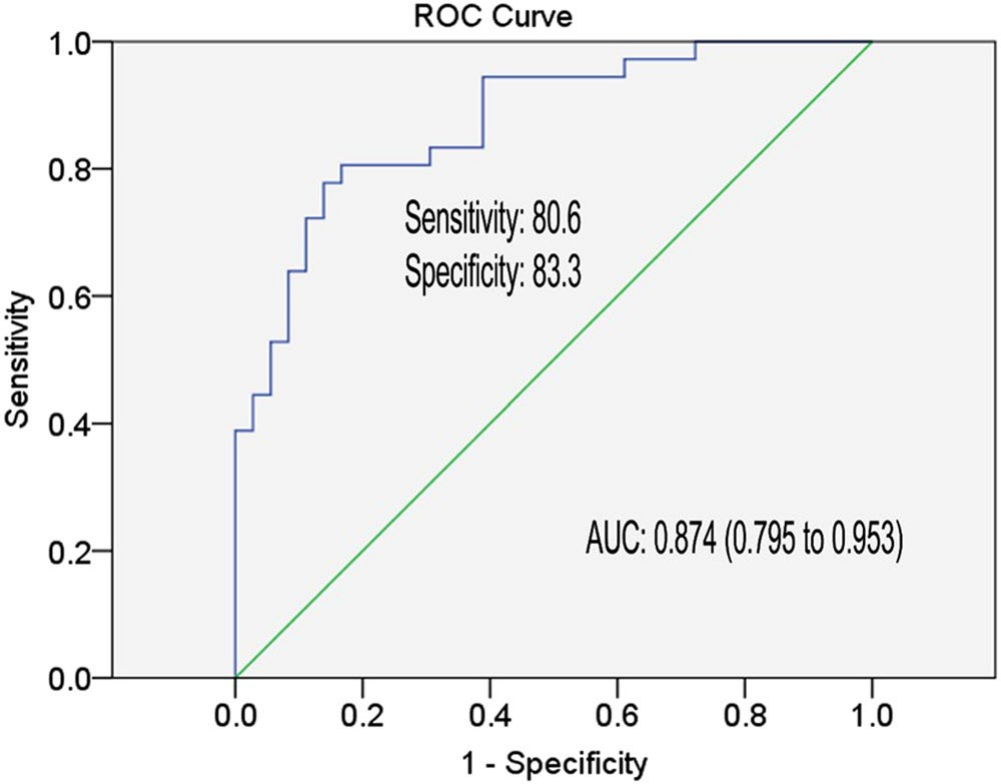
Receiver operating characteristic curve analysis for the prediction of EVR of CHB patients with IFN-α therapy. Area under curve (AUC) depicts the estimation of prediction value for the levels of selected miRNAs and ALT in the training phase. The green line represents the reference line of AUC = 0.5.

Next, miR-22, miR-210, miR-1260a, identified in plasma of CHB patients in the training phase, in conjunction with baseline levels of ALT were used to construct the logistic regression model, which could be used to differentiate responders to IFN-α therapy. Stepwise logistic regression analysis led to the final identification of miR-22, miR-210 and ALT as independent constituents of the predictive model, logit (p) = −14.856 + 0.012 × ALT − 0.519 × miR-22 + 3.831 × miR-210. The AUC, as determined by ROC curve analysis, in the training phase yielded a value of 0.874 (95% CI, 0.795 to 0.953; sensitivity = 80.6%, specificity = 83.3%), (Fig. 3).

**Figure 4:**
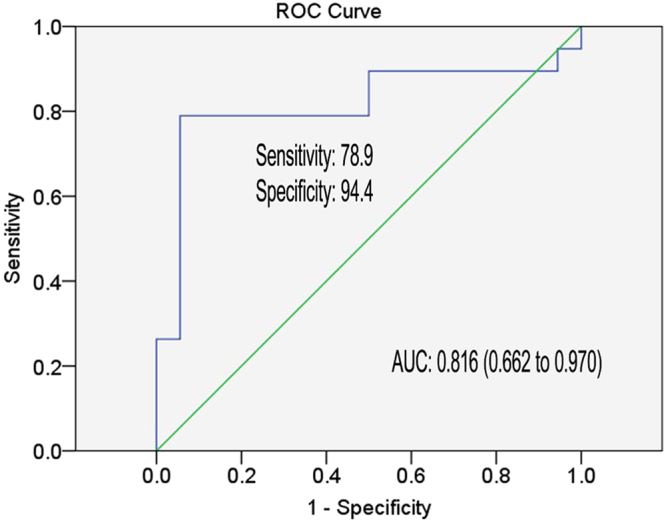
Receiver operating characteristic curve analysis for the prediction of EVR of CHB patients with IFN-α therapy. Area under curve (AUC) depicts the estimation of prediction value for the levels of selected miRNAs and ALT in the validation phase. The green line represents the reference line of AUC = 0.5.

We next tested our model through a validation phase. The established prediction algorithm was applied to additional 37 cases receiving IFN-α therapy, and yielded an AUC of 0.816 (95% CI, 0.662 to 0.970; sensitivity = 78.9%, specificity = 94.4%), (Fig. 4). Taken together, the established prediction model consisting of miR-22, miR-210 and ALT provided meaningful prediction accuracy in both the training and validation phases.

Since the baseline level of ALT represents an important factor for the prognosis of CHB patients, we stratified the whole cohort (n = 112) according to the baseline levels of ALT (cutoff = 100 U/L) and tested the performance of the established scoring model. In the elevated ALT group (>100 U/L, n = 58), the AUC of the predictive factors panel was 0.887 (95% CI, 0.791 to 982; sensitivity = 82.1%, specificity = 89.5%), (Supplementary Figure 2A), and in the low ALT group (≤100 U/L, n = 54), the AUC of the predictive factors panel achieved value of 0.857 (95% CI, 0.747 to 0.966; sensitivity = 87.5%, specificity = 73.5%), (Supplementary Figure 2B). These results indicated that our predictive scoring model exhibited a consistent performance irrespective of baseline ALT levels.

### Performance of the model in predicting sustained virological response

**Figure 5:**
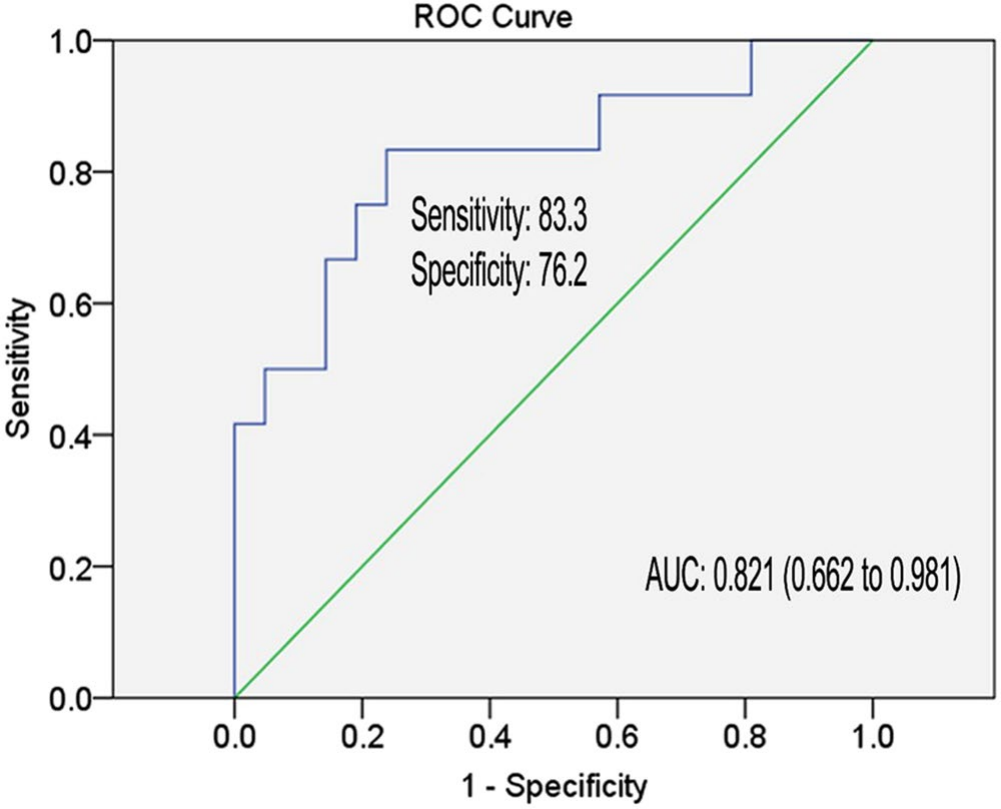
Receiver operating characteristic curve analysis for the prediction of SVR of CHB patients with IFN-α therapy. Area under curve (AUC) depicts the estimation of prediction value for the levels of selected miRNAs and ALT in the long-term prognosis. The green line represents the reference line of AUC = 0.5.

In order to ensure the clinical significance of our scoring model in forecasting the long-term outcome of IFN-α treatment of CHB patients, we correlated model’s prediction with the sustained virological response. For this, 35 patients were amenable for SVR analysis while the other 77 patients, initially enrolled in the study, were excluded due to other antiviral medications within 48 weeks of treatment or because of withdrawal due to side-effects etc. Among the 35 patients 12 achieved SVR while 23 were defined as N-SVR (Table 3). We found that all of the 35 patients were HBeAg positive at baseline and the basic characteristics of SVR and N-SVR were comparable. Finally, the AUC of the model achieved value of 0.821 (95% CI, 0.662 to 0.981; sensitivity = 83.3%, specificity = 76.2%), (Fig. 5) in predicting the sustained virological response of CHB patients.

**Table 3:** The comparison of baseline and end-of-therapy clinical variables between N-SVR and SVR at 12 months.

Variables	N-SVR	SVR	P value
**Number (total, %)**	23 (35, 65.7%)	12 (35, 34.3%)	
**Age (year)**			0.5769
median	28	29	
range	26–32.5	26–30	
**Gender**			0.4769※
Male (%)	15 (65.2)	6 (50)	
Female (%)	8 (34.8)	6 (50)	
**Genotype**			0.2469※
B type (%)	7 (30.5)	6 (50)	
C type (%)	14 (60.9)	4 (33.4)	
D type (%)	1 (4.3)	1 (8.3)	
Missing (%)	1 (4.3)	1 (8.3)	
**Baseline clinical variables**			
**ALT (U/L)**			0.7789
median	90	91.5	
range	70–125	77.5–122.3	
**HBV DNA (log**_**10**_ **copies/ml)**			0.6495
median	7.2	6.8	
range	6.2–8.0	6.19–7.53	
**HBsAg (log**_**10**_ **COI)**			0.5235
median	4.14	3.87	
range	3.70–4.73	3.51–4.39	
**HBeAg (log**_**10**_ **COI)**			0.0391
median	3.3	1.7	
range	2.05–3.68	0.16–3.38	
**End of therapy clinical variables**			
**HBV DNA (log**_**10**_ **copies/ml)**			<0.0001
median	4.5	2.7	
range	3.35–6.51	2.69–2.87	
**HBsAg (log**_**10**_ ** IU/ml)**			0.0043
median	3.86	2.6	
range	3.32–4.30	1.76–3.44	
**HBeAg (log**_**10**_ **COI)**			<0.0001
median	1.53	−0.44	
range	0.64–3.36	−0.48–−0.39	

Abbreviations: IU = International Unit; COI = Cut Off Index; N-SVR = Non Sustained Virological Response, SVR = Sustained Virological Response; ALT = serum alanine amino transferase; DNA = deoxyribonucleic acid; HBeAg = hepatitis B e antigen. The levels of HBV DNA, HBsAg and HBeAg were log10 transformed. Only genotype frequency of B and C were analyzed. P value: Comparison between N-SVR and SVR (Mann Whitney test); ※Fisher’s exact test.

Additionally, according to the univariate logistic regression analysis on independent factors associated with SVR, we found that the prediction model and, in particularly miR-210 highly associated with SVR (data not show). While, other clinical parameters including HBV genotype (B vs C), HBV DNA, HBsAg and ALT did not show statistical significance in our study (data not show).

In order to better understand the relationships between candidate miRNAs with clinical and virological variables, we also analyzed the correlation between the levels of miR-22 and miR-210 with ALT, HBV DNA, HBsAg and HBeAg. Our results demonstrated that the levels of miR-22 and miR-210 both showed significant correlation with HBV DNA. In addition, the levels of miR-22 also showed significant correlation with ALT. However, neither miR-210 nor miR-22 show any correlation with HBsAg and HBeAg (Supplementary Table 3).

In summary, the model which we developed can be used in clinic as a reliable predictor for both the sustained virological response in addition to early virological response.

## Discussion

As an important therapy for chronic hepatitis B, IFN-α has certain advantages for HBeAg seroconversion, off-treatment sustained response and HBsAg seroconversion^3,10^. However, considering inconvenient dosing schedule and potential side-effects, the use of IFN-α has certain risks and challenges. Therefore, to improve the overall treatment success rate, it is necessary to develop a practical estimation for the likelihood of the sustained response to IFN-α treatment^13^.

Circulating miRNAs were described as important predictors in differentiating stages of liver diseases^14,15,27^. For example, liver specific miR-122 was up-regulated in alcohol-induced hepatocyte impairment, while high level of miR-155 could predict the inflammatory liver injury^14^. A significant increase in the levels of liver-enriched miR-122 and miR-192 were also observed in patients following acetaminophen-induced acute liver injury^17^. Besides, Zhou *et al*., established a plasma miRNAs panel that could be used, with high prediction accuracy (AUC = 0.864), in diagnosis of early-stage hepatocellular carcinoma (HCC). The predictive effect of this plasma miRNAs panel was validated in an independent cohort with consistent performance^28^.

In the current study we simplified and improved the predictive value of miRNAs model by examining the expression of 15 miRNAs along with various clinical parameters in 112 CHB patients who were divided into the training or validation groups. The stepwise regression analysis of results from the training group led to an optimized scoring model, which included miR-22, miR-210 and ALT, showing high prediction accuracy in both the training (AUC = 0.874) and validation (AUC = 0.816) groups. Besides, this scoring model also performed well in predicting the sustained virological response of CHB patients to IFN-α treatment.

The clinical indicators of CHB patients – such as baseline ALT levels, HBV DNA levels and HBV genotypes, age, gender, etc. were considered as predictors of the interferon alpha therapy outcome^9,12^. For example, in HBeAg–positive CHB patients, the high baseline levels of ALT and low level of HBV DNA (<2 × 10^8^ IU/ml) could serve as independent factors that predict higher response rate to conventional IFN-α treatment^12^. HBV genotype is also an important predictor for the curative effect of IFN-α treatment. Compared with the genotype D and C, patients infected with HBV genotype A were more likely to achieve response, determined as HBeAg loss, following IFN-α therapy^4^. In terms of HBeAg clearance, Asian patients infected with HBV genotype B had better response to conventional IFN-α than those infected with genotype C^9^. The decline of HBsAg levels induced by IFN-α treatment of HBeAg-positive CHB patients may be considered as a biomarker to predict success of the therapy^29^. Despite of this progress, the predictive value of these clinical parameters is still limited and need to be improved with novel non-invasive markers^12^.

In this study, we also considered the predictive value of prognosis-associated clinical variables, including ALT, HBV DNA, HBsAg, HBV genotype etc. Except for ALT, other clinical parameters did not reach statistical significance in our study. Furthermore, we preformed the univariate logistic regression analysis to compare the significance of the miRNA predictor with clinical variables. We found that miRNA signature showed higher association with SVR than other clinical parameters. Nevertheless, we recognize that the limited cohort size may influence the performance of clinical variables in our study.

It has been demonstrated that miRNAs, known post-transcriptional regulator, are involved in the development of liver fibrosis and cirrhosis^30^. Unfortunately, in this study there was insufficient information as to the stage of liver fibrosis in all the patients. Our attempts to correlate miRNAs with development of liver cirrhosis in 22 patients did not yield meaningful information.

MiR-22 and miR-210 signature was highly associated with SVR of CHB patients. These two miRNAs have been reported to play a role in HBV infection and immune response, although the exact mechanism is not well understood.

The levels of miR-22 were upregulated at least 1.5 fold in serum of HBV-infected patients^23^. In the development of cirrhosis, miR-22 was shown to play a positive role by binding to the 3′-UTR to downregulate BMP7 expression^31^. While, in HBV-related HCC, it was reported that miR-22 targets CDKN1A to suppress the progression^32^. Levels of miR-210 were shown to fluctuate in pathological conditions associated with liver diseases. For example, expression of miR-210 could be elevated as a response to hypoxia-associated liver inflammation, thus serum miR-210 levels may indicate the severity of hepatitis^33^. Besides, miR-210 was reported to suppress HBV replication by targeting pre-S1 region in HBV genome^25^. In murine macrophages, LPS-induced expression of miR-210 was shown to suppress production of proinflammatory cytokines via a negative regulatory feedback involving NF-кB1^34^.

In conclusion, we developed a simplified scoring model for predicting the outcome of IFN-α treatment of CHB patients. The model encompasses three factors, miR-22, miR-210 and ALT, that can distinguish EVR and SVR from non-response among CHB patients with high accuracy and over extended time post treatment. Hence, this predictive factors panel may have a considerable clinical value by providing more meaningful guidance for clinicians to identify CHB patients who would respond favorably to IFN-α treatment. Further assay standardization and independent validation in larger cohorts are under way.

## Patients and Methods

### Patients

A total of 112 CHB patients who underwent IFN-α treatment at Shanghai Public Health Clinical Center, Huashan Hospital and Ruijin Hospital were enrolled. The patients were administered with either Peg-IFN-alfa-2a (180 µg/week, 81 patients), or were given Peg-IFN-alfa-2b (based on weight at 1 µg/kg once a week, 31 patients). Among the enrolled CHB patients, 98 patients (87.5%) were HBeAg-positive while 14 patients (12.5%) were HBeAg-negative. The following inclusion criteria were used: no antiviral therapy within 6 months of initiation of the study; viral loads over 10^4^ copies per milliliter (copies/ml); positivity for HBsAg for longer than 6 months; abnormal plasma ALT levels (>1.0 ULN). Further exclusion criteria included: co-infection with hepatitis A virus (HAV), hepatitis C virus (HCV), hepatitis E virus (HEV), human immunodeficiency virus (HIV) or decompensated cirrhosis; the presence of autoimmune diseases; evidence of liver comorbidities consequent to alcohol, drugs and autoimmune disease; serious concurrent medical illnesses (e.g. malignancies, severe cardiopulmonary disease, psychiatric disorders, uncontrolled diabetes mellitus); pregnancy or nursing; or withdrawal from this study because of treatment mediated side effects. The treatment outcome of each patient at different time point was defined as follows: early virological response (EVR) indicated a ≥2 log10 decrease of viral load and non-early virological response (N-EVR) indicated a <2 log10 decrease of viral load at the 12th week after starting treatment. Sustained virological response (SVR) indicated HBeAg loss and <2.0 × 10^3^ IU/ml of viral load at the end of the 48-week IFN-α therapy whereas the rest was defined as non-sustained virological response (N-SVR)^10^. The total number of patients that were amenable to SVR analysis was 35 due to the exclusion of subjects who took additional nucleos(t)ide analogs suggested by the physicians or who voluntarily withdrew from IFN-α therapy because of side-effects. Informed consent was obtained from each patient included in the study. The study conforms to the ethical guidelines of the 1975 Declaration of Helsinki and was approved by the ethics committee of Shanghai Public Health Clinical Center.

### Study design

This study was a retrospective study and the CHB patients who received IFN-α treatment were randomly assigned into our cohort study. The basic clinical characteristics of enrolled patients are presented in Table 1. This study was carried out in two phases, namely, the training and the validation phase as depicted in Fig. 1. Patients’ plasma samples were tested for the baseline levels of candidate miRNAs by RT-PCR. The candidate miRNAs included 9 miRNAs, i.e., has-let-7a, has-let-7f, has-miR-22, has-miR-30a, has-miR-106b, has-miR-638, has-miR-1224-5p, has-miR-1281, and has-miR-1290, which were identified via microarray analyses in our previous study^7^, plus 6 additional miRNAs, namely has-miR-99a, has-miR-122, has-miR-199a, has-miR-210, has-miR-1260a, has-miR-4284, which had been linked to HBV replication, life cycle or associated with antiviral immunity^22–25^. MiRNAs that were differentially expressed in N-EVR versus EVR groups were identified by Mann-Whitney test. A stepwise multivariate logistic regression analysis was performed to formulate a prediction scoring model whose performance was evaluated by receiver operating characteristic (ROC) curve analysis.

In the validation phase 37 patients were included. The levels of candidate miRNAs were also quantified and a score was calculated for each patient using the parameters generated in the training phase. In order to test whether the prediction model was repeatable, a ROC curve analysis based on the calculated probabilities was performed.

### Laboratory measurements

Fasting plasma was collected from all participants before the initiation of IFN-α treatment. Plasma samples were analyzed for clinical indicators, including the levels of HBsAg, HBeAg, ALT, HBV DNA and HBV genotype. The expression levels of HBsAg and HBeAg were measured by Elecsys HBsAg II assay (Roche Diagnostics, Mannheim, Germany) and Elecsys HBeAg assay (Roche Diagnostics, Mannheim, Germany), respectively, both using an electro-chemiluminescence instrument (E601, Roche). Plasma ALT levels were measured with an automatic biochemical analyzer (P800, Roche). HBV DNA was quantified by a quantitative PCR assay (Qiagen, Shenzhen, China) according to the recommended protocol. HBV genotyping was performed using type A through F genotype specific PCR primers (Biosune, Shanghai, China) .

### MiRNA extraction

To obtain fresh plasma, patients’ blood samples were first centrifuged at 200 × g for 15 min. Plasma samples were then centrifuged at 2,000 × g for 20 min at 4 °C and the supernatants were again centrifuged at 10,000 × g for 30 min followed by a final centrifugation at 14,000 × g for 10 min at 4 °C to completely remove cell debris. Total RNA was extracted from 100 μl plasma using TRIzol-LS (Pufei, Shanghai, China) according to the recommended protocol. Cel-miR-39 (RiboBio, Guangzhou, China) served as an external control in all assays.

### Reverse transcription and quantitative PCR

Following precipitation, RNA was re-suspended in RNase-free water together with mixed specific Bulge-Loop RT primers (RiboBio, Guangzhou, China) and incubated at 70 °C for 10 min. Then the mixture was reverse-transcribed using Multiscribe reverse transcriptase (Applied Biosystems, Foster City, CA, USA) in RT reaction (16 °C, 30 min; 42 °C, 1 h; 85 °C, 5 min; 4 °C∞).

Real-time PCR reactions were performed using 2 x SYBR Green Master Mix (TOYOBO, Osaka, Japan) according to the manufacturer’s directions in a ViiA7 Real-Time PCR system (Applied Biosystems, Foster City, CA, USA). The reactions, running in duplicate, were carried out in 384-well plates at 95 °C for 1 min, followed by 40 cycles of 95 °C for 10 s and 60 °C for 30 s. Following 40 cycles of amplification, melting curve analysis was performed in each run. The relative amount of miRNAs was calculated using the equation 2−△Ct by the standards that the CT value of 30 cycles as equal to relative levels of 1. All the relative expression levels of miRNAs were log10 transformed.

Plasma samples were subjected to two freeze-thaw cycles over one month and the expression levels of miRNAs were largely unchanged following freeze-thaw at −80 °C (Data not shown). The stability of plasma miRNAs and the reproducibility of the method were confirmed.

### Statistical analysis

The statistical differences of clinical characteristics and miRNA expression levels between N-EVR and EVR groups were compared using Mann-Whitney test. A stepwise logistic regression with defined covariates of interest was used to determine the capacity of pre-treatment factors in predicting the EVR of IFN-α therapy in CHB patients. The final predictors and parameters of the logistic regression were fitted with the maximum likelihood procedure. The area under the ROC curve (AUC) was calculated for these variables, and AUC values closer to 1 indicated higher prediction accuracy. Data was analyzed by using the Graph Pad Prism (version 5.01).

### Data Availability

All data generated or analysed during this study are included in this published article (and its Supplementary Information files).

## Electronic supplementary material

Supplementary information
