# Supplementary material for: Circulating miR-210 and miR-22 combined with ALT predict the virological response to interferon-alpha therapy of CHB patients
Source: Sci Rep. 2017 Nov 15;7:15658. doi: 10.1038/s41598-017-15594-0 (PMC5688172; doi:10.1038/s41598-017-15594-0)
Supplement: Supplementary file 1 — Supplementary information [file 41598_2017_15594_MOESM1_ESM.pdf]

Circulating miR-210 and miR-22 combined with ALT predict the virological response to interferon-alpha therapy of CHB patients

Jin Li[1,2#], Xiaonan Zhang[1,2#], Liang Chen[3], Zhanqing Zhang[3], Jiming Zhang[4], Weixia Wang[1,2], Min Wu[1,2], Bisheng Shi[1,2], Xinxin Zhang[5], Maya Kozlowski[1,2], Yunwen Hu[1*], and Zhenghong Yuan[1,2*]

1 Research Unit, Shanghai Public Health Clinical Center, Fudan University, Shanghai, China.

2 Key Laboratory of Medical Molecular Virology at the School of Basic Medical Sciences, Shanghai Medical College, Fudan University, Shanghai, China.

3 Department of Hepatology, Shanghai Public Health Clinical Center, Fudan University, Shanghai, China.

4 Department of Infectious Diseases, Huashan Hospital, Fudan University, Shanghai, China.

5 Institute of Infectious and Respiratory Diseases, School of Medicine, Shanghai Jiaotong University, Ruijin Hospital, Shanghai, China.

Correspondence: Zhenghong Yuan, Key Laboratory Medical Molecular Virology, Shanghai Medical College, Fudan University, 138 Yixueyuan Road,

Shanghai 200032, China; Tel: (+86-21) 54237669; Fax: (+86-21) 64227201; e-mail: zhyuan@shaphc.org. Yunwen Hu, Research Unit, Shanghai Public Health Clinical Center, Fudan University, 2901 Caolang Road, Jin Shan District, Shanghai 201508, China; Tel: (+86-21) 37990333-6514; Fax: (+86-21) 57249023; e-mail: ywhu0117@126.com.

[#]These two authors contributed equally to this work.

*Y.H. and Z.Y. are joint senior authors, contributing equally to the direction of this study.

This publication is dedicated to the memory of Professor Yunwen Hu who suddenly passed away during the preparation of the manuscript. Professor Yunwen Hu was an invaluable mentor and inspiration for this study.

Figure legends

Supplementary Figure 1. Comparison of clinical and virological parameters of CHB patients receiving IFN-α treatment in the training and validation phases. (A-D) The levels of ALT (p=0.0065), HBV DNA (p=0.1670), HBsAg (p=0.4334) and HBeAg (p=0.8211) were compared between EVR and N-EVR groups in the training phase. (E-H) The levels of ALT (p<0.0001), HBV DNA (p=1.000), HBsAg (p=0.1176) and HBeAg (p=0.2545) were compared between EVR and N-EVR groups in the validation phase. The levels of HBV DNA, HBsAg and HBeAg were log10 transformed. The lines in boxes represent median values, and the deviation bars represent min to max values (Mann Whitney test).

Supplementary Figure 2. Receiver operating characteristic curve analysis for the prediction of IFN-α therapy outcome in the whole cohort (n = 112) stratified by different ALT levels. (A) Area under the curve (AUC) estimation for the miRNA panel in the elevated ALT (>100U/L) group. (B) Area under the curve (AUC) estimation for the miRNA panel in the low ALT (≤100 U/L) group.

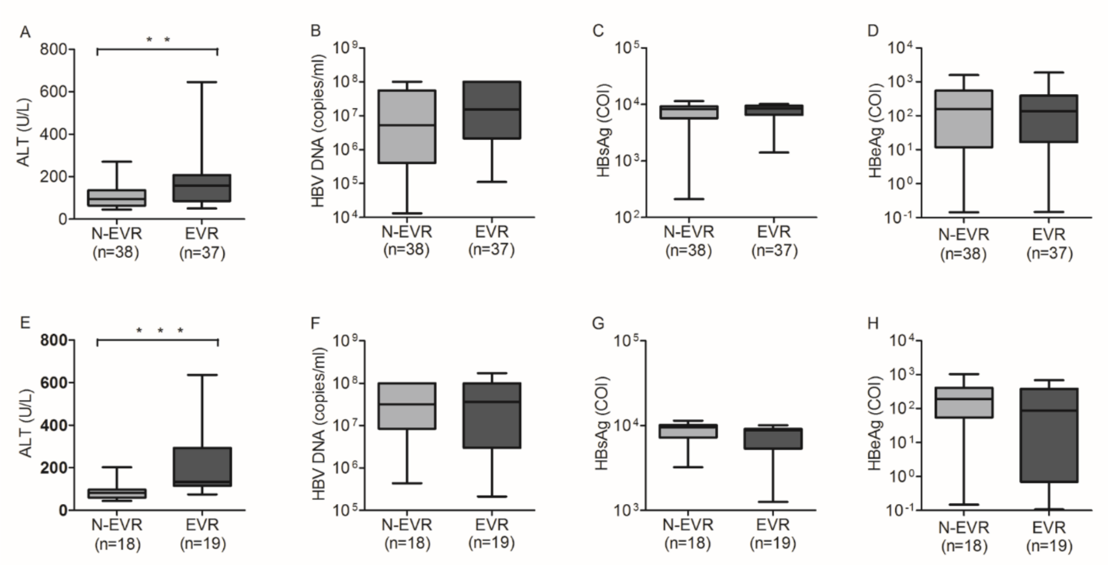
A
B
C
D
E
F
G
H
ALT (U/L)
HBV DNA (copies/ml)
HBsAg (COI)
HBeAg (COI)
N-EVR (n=38)
EVR (n=37)
N-EVR (n=18)
EVR (n=19)
* *
* * *

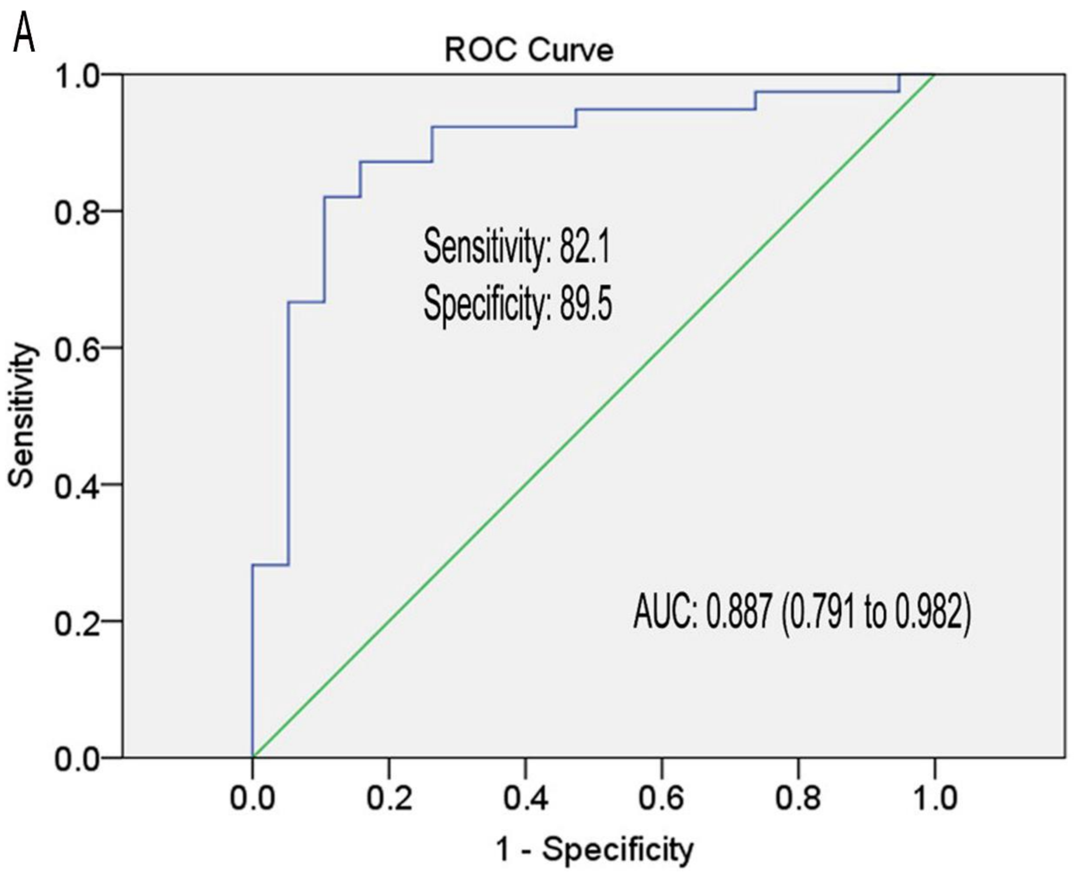
A
ROC Curve
Sensitivity: 82.1
Specificity: 89.5
AUC: 0.887 (0.791 to 0.982)
Sensitivity
1 - Specificity

B

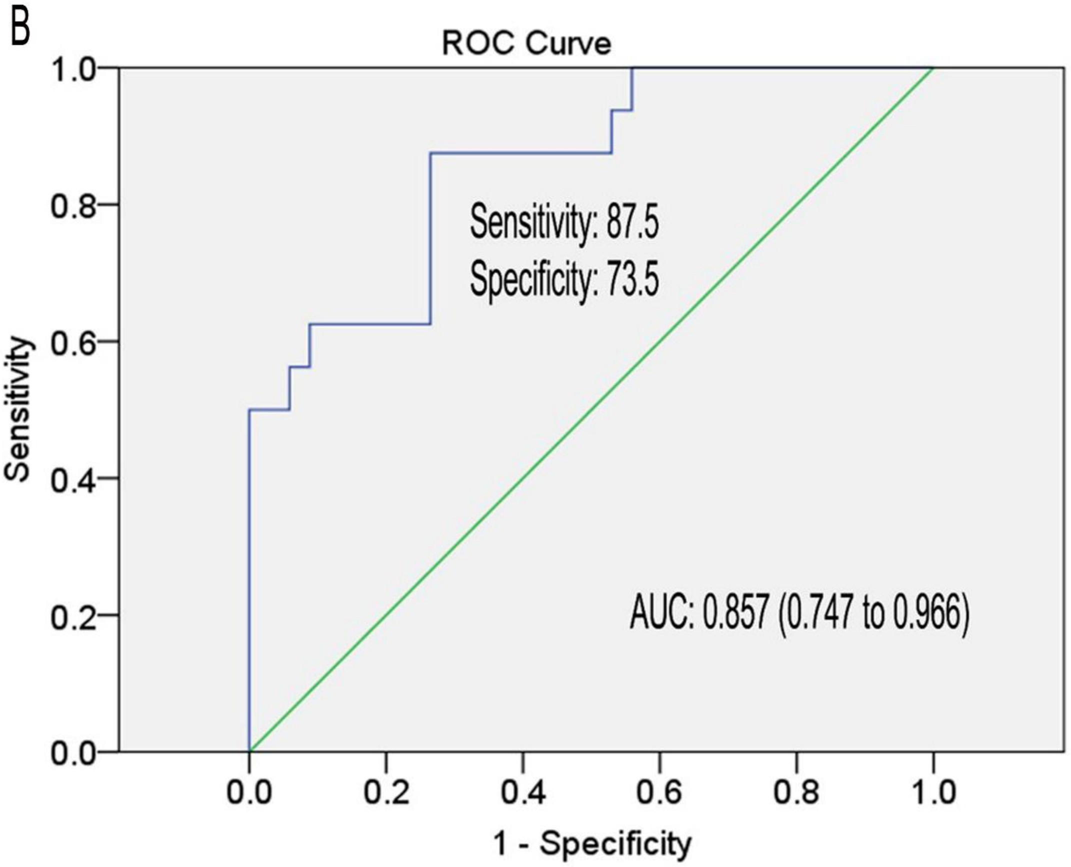
ROC Curve
Sensitivity: 87.5
Specificity: 73.5
AUC: 0.857 (0.747 to 0.966)
Sensitivity
1.0
0.8
0.6
0.4
0.2
0.0
0.0
0.2
0.4
0.6
0.8
1.0
1 - Specificity

microRNA	Cellular targets	Reported biological functions
let-7f	PRR signaling	Biomarkers of HCC<br>Induce of immune tolerance
miR-22	CDKN1A	Tumor suppressor- HBV-related HCC
miR-30a	SNAI1	Reduce invasion and metastasis of HCC
miR-99a	FGFC3<br>AGO2/miR-21/PTEN	Tumor suppressor- bladder cancer<br>Control tumor growth in HCC
miR-199a	HBV S protein<br>HBV polymerase	Suppress HBV replication
miR-210	pre-S1 region<br>HBV polymerase	Suppress HBV replication
miR-1224	Sp1	Negative regulators of TNF-a<br>Involved in LPS-mediated<br>Inflammatory responses
miR-1260a	Unknown	Inhibit HBV replication
miR-4284	Unknown	Antitumor<br>Inhibit HBV replication

Supplement Table 1. The targets and functions of candidate microRNAs.

Abbreviations: HCC= Hepatocellular carcinoma; PRR= Pattern recognition receptor; CDKN1A= Cyclin Dependent Kinase Inhibitor 1A; SNAI1=Snail; FGFC3= Fungi fibrinolytic compound 3; AGO2= Argonaute 2; PTEN= phosphatase and tensin homolog deleted on chromosome ten; Sp1= Specificity protein-1.

microRNA	N-EVR group(n=38)	EVR group(n=37)	P value
has-let-7a	#0.20 (-1.23 – 1.80)	0.19 (-1.59 – 2.19)	0.7147
has-let-7f	-0.92 (-2.85 – 0.54)	-1.07 (-2.76 – 0.81)	0.3414
has-miR-22	1.43 (0.25 – 2.07)	1.88 (-0.68 – 2.41)	< 0.0001
has-miR-30a	0.26 (-1.18 – 1.56)	0.17 (-1.88 – 1.70)	0.2459
has-miR-99a	-0.19 (-2.27 – 1.84)	-0.21 (-2.59 – 2.12)	0.8033
has-miR-106b	0.06 (-0.87 – 1.03)	0.34 (-1.23 – 1.54)	0.0133
has-miR-122	2.09 (0.64 – 3.83)	2.06 (-0.42 – 3.85)	0.8198
has-miR-199a	0.29 (-0.96 – 1.56)	-0.03 (-0.69 – 2.39)	0.4614
has-miR-210	3.32 (2.50 – 4.34)	4.03 (3.14 – 4.34)	< 0.0001
has-miR-638	2.57 (1.96 – 3.19)	2.39 (2.01 – 2.93)	0.0018
has-miR-1224	0.04 (-0.88 – 0.84)	0.45 (-0.41 – 0.79)	< 0.0001
has-miR-1260a	4.47 (4.13 – 4.68)	4.58 (4.32 – 4.71)	< 0.0001
has-miR-1281	0.54 (-0.12 – 1.05)	0.79 (-0.70 – 1.32)	0.0004
has-miR-1290	1.61 (0.57 – 3.20)	1.82 (-0.90 – 3.25)	0.0759
has-miR-4284	-1.49 (-1.99 – -0.86)	-0.98 (-1.90 – -0.30)	< 0.0001

Supplementary Table 2. Relative expression of 15 plasma microRNAs in the training phase at baseline.

Abbreviations: EVR=Early Virological Response, N-EVR=Non Early Virological Response; The levels of miRNAs were log10 transformed; P value: Comparison between N-EVR and EVR groups at baseline (Mann Whitney test); #Median (Minimum - Maximum).

microRNAs	Variables	Correlation coefficients	P values
**miR-22**	ALT	0.2992	0.0017
	HBV DNA	0.4117	<0.0001
	HBsAg	0.0728	0.4496
	HBeAg	0.0477	0.6208
	miR-210	0.6857	<0.0001
**miR-210**	ALT	0.0743	0.4446
	HBV DNA	0.3248	0.0005
	HBsAg	0.0688	0.4748
	HBeAg	-0.0233	0.8090
	miR-22	0.6857	<0.0001

Supplementary Table 3. The correlation between the levels of miR-22 and miR-210 with biochemical and virological markers.

Abbreviations: ALT=serum alanine amino transferase; DNA= deoxyribonucleic acid; HBsAg=hepatitis B surface antigen; HBeAg=hepatitis B e antigen; Correlation coefficients and P values: Nonparametric correlation (Spearman).
